# Observations on the Medical Treatment of General Washington's Last Illness

**Published:** 1800-05

**Authors:** John Reid

**Affiliations:** Physician of the Finsbury Dispensary, &c.


### *Obſervations on the Medical Treatment of General Waſhington's laſt Illneſs.* *By* JOHN REID, M. D. Phyſician of the Finſbury Diſpenſary, &c.

IN reading the official report of the death of General Waſhington, as ſtated in the news-papers, &c. I ſhould imagine, there were few medical perſons who did not feel aſtoniſhment at the very extraordinary manner in which that great man was treated by his phyſicians, during his laſt and fatal indiſpoſition.

Some time in the night of the 13th of December, it is ſaid, that the General was ſeized by a diſeaſe, called the Cynanche Trachealis.

During the ſame night, he ſent for a bleeder, who took from him twelve or fourteen ounces of blood.

The next morning, a phyſician was ſent for, who arrived at Mount Vernon, at eleven o'clock; when, imagining danger in the caſe, he adviſed the calling in of two conſulting phyſicians.

In the interval, however, he thought proper to employ, in ſpite of the twelve or fourteen ounces that had already been expended, two copious bleedings. Now, when we conſider that theſe are called copious, and the other is not noticed as ſuch, and alſo the indifference with which a future moſt copious ous bleeding is afterwards mentioned, we may presume, that each of these was twenty-five, or twenty, ounces at least.

After this, "two moderate doses of calomel were administered." I know not exactly what a moderate American dose of calomel may be; but if, as it is fair to presume, it be in proportion to the bleedings, we may conclude, that it was at least very considerable.

Upon the arrival of the first of the consulting physicians, it was agreed, that as there were no signs of accumulation in the bronchial vessels of the lungs, they should try another bleeding.

Now this appears to be perfectly inexplicable.

As there were at present, no signs of accumulation in the bronchial vessels of the lungs, they were driven to another bleeding. Hence, it would seem, that this last bleeding was to *produce* an accumulation in the bronchial vessels of the lungs. There was great difficulty of breathing, great inflammation; but as there was, as yet, no accumulation in the lungs, they were determined to induce that also; and, as a likely mean of inducing it, had recourse to a most extravagant effusion of blood. This is not an unfair interpretation of their words; but it could not have been their real meaning: their real meaning it is impossible to discover. In addition to all the previous venefections, thirty-two ounces are now drawn! The medical reader will not be surprised to find that this was unattended by any apparent alleviation of the disease.

In the next place, vapors of vinegar and water are frequently inhaled. Two doses of calomel were already given; but this is not deemed sufficient, ten grains of calomel are added: Nor is even this sufficient; repeated doses of emetic tartar, amounting, in all, to five or six grains, are next administered. It is said, "the powers of life now seemed to yield to the force of the disorder." To many it may appear, that the yielding of the vital principle, in these circumstances, was not *altogether* owing to the force of the disorder.

The patient, lying in this feeble and nearly exhausted state, is to be still farther tormented: Blisters are next applied to his extremities, together with a cataplasm of bran and vinegar to his throat.

It is observed, that "speaking, which was painful from the beginning, now became scarcely practicable." When we reflect upon that extreme degree of weakness to which the patient must, by this time, have been reduced, and that he had both a blister and a cataplasm of bran and vinegar at his throat, can we wonder that speaking would be scarcely practicable? Respiration grew more and more contracted and imperfect until til after eleven o'clock on the Saturday night, when he expired without a ſtruggle.

Think of a man being, within the brief ſpace of little more than twelve hours, deprived of eighty or perhaps ninety ounces of blood; afterwards ſwallowing two moderate American doſes of calomel, which were accompanied by an injection; then five grains of calomel, and five or ſix grains of emetic tartar; vapors of vinegar and water frequently inhaled; bliſters applied to his extremities; a cataplaſm of bran and vinegar to his throat, upon which a bliſter had been already fixed: Is it ſurpriſing that, when thus treated, the afflicted General, after various ineffectual ſtruggles for utterance, at length articulated a deſire that he might be allowed to die without interruption!

To have reſiſted the fatal operation of ſuch Herculean remedies, one ſhould imagine that this venerable old man ought at leaſt to have retained the vigor of his earlieſt youth.

A Britiſh phyſician may be deemed not competent to aſcertain the propriety of trans-atlantic practice; the current of blood, in the inhabitants of the new world, may bear ſome proportion to the current of its rivers; in that caſe, the medical treatment ought likewiſe to be conducted upon a larger ſcale.

But this is a ſubject not proper for levity; it is a ſerious and ſolemn ſubject; and it is on that account that I have been induced to make the few preceding obſervations.

*London, April 21, 1800.* J. REID.

CRITICAL

