# Errata

**Published:** 1800-05

**Authors:** 


					jP* 475? * It ">r fa1 gw?* ot caloatfii read grams-? caiwaj^

				

